# Growth Cone Tctp Is Dynamically Regulated by Guidance Cues

**DOI:** 10.3389/fnmol.2018.00399

**Published:** 2018-11-06

**Authors:** Cláudio Gouveia Roque, Christine E. Holt

**Affiliations:** ^1^Department of Physiology, Development and Neuroscience, University of Cambridge, Cambridge, United Kingdom; ^2^Doctoral Programme in Experimental Biology and Biomedicine, Center for Neuroscience and Cell Biology, University of Coimbra, Coimbra, Portugal

**Keywords:** Tctp, mTORC, Netrin-1, Ephrin-A1, axon guidance, retinal ganglion cell, growth cone, protein translation

## Abstract

Translationally controlled tumor protein (Tctp) contributes to retinal circuitry formation by promoting axon growth and guidance, but it remains unknown to what extent axonal Tctp specifically influences axon development programs. Various genome-wide profiling studies have ranked *tctp* transcripts among the most enriched in the axonal compartment of distinct neuronal populations, including embryonic retinal ganglion cells (RGCs), suggesting its expression can be regulated locally and that this may be important during development. Here, we report that growth cone Tctp levels change rapidly in response to Netrin-1 and Ephrin-A1, two guidance cues encountered by navigating RGC growth cones. This regulation is opposite in effect, as we observed protein synthesis- and mTORC1-dependent increases in growth cone Tctp levels after acute treatment with Netrin-1, but a decline upon exposure to Ephrin-A1, an inhibitor of mTORC1. Live imaging with translation reporters further showed that Netrin-1-induced synthesis of Tctp in growth cones is driven by a short 3′untranslated region (3′UTR) *tctp* mRNA isoform. However, acute inhibition of *de novo* Tctp synthesis in axons did not perturb the advance of retinal projections through the optic tract *in vivo*, indicating that locally produced Tctp is not necessary for normal axon growth and guidance.

## Introduction

Axon guidance is informed by successive molecular signposts—guidance cues—in the embryonic nervous system that are continuously integrated by the growth cone, a sensory structure at the tip of developing axons. Studies spanning the past three decades have revealed that a certain degree of functional autonomy is held by this cellular outpost, perhaps best illustrated by the demonstration that axons separated from their cell bodies can still navigate correctly *in vivo* (Harris et al., 1987). This operational independence is, understandably, essential for pathfinding growth cones to respond rapidly and precisely to their ever-changing environment. It is now clear that this flexibility arises in part from the swift regulation of the axonal proteome through local protein production and degradation mechanisms, as interference with axonal proteostasis disrupts normal axon growth and guidance (Campbell and Holt, 2001; Hamilton and Zito, 2013; Leung et al., 2013; Deglincerti et al., 2015).

We have previously documented that translationally controlled tumor protein (Tctp; gene symbol: *tpt1*) cell-autonomously contributes to the precise and timely development of axon projections during the wiring of *Xenopus* retinal circuitry. In our original study, a combination of central nervous system-wide knockdown approaches revealed that Tctp regulates axonal growth and guidance, and leads to compromised mitochondrial operation in axons (Roque et al., 2016). In line with these findings, research on Tctp has generally defined it as a survival and growth-promoting factor (Kamath et al., 2003; Hsu et al., 2007; Zhang et al., 2008; Brioudes et al., 2010; Koziol and Gurdon, 2012; Jojic et al., 2018). The earliest *in vivo* clue linking Tctp to growth programs came from a large-scale RNAi screen in *C. elegans* documenting slow-growth phenotypes upon Tctp depletion (Kamath et al., 2003), a finding later independently confirmed in flies and rodents (Hsu et al., 2007; Susini et al., 2008). Curiously, overexpression of Tctp *in vitro* also results in impaired cell growth (Gachet et al., 1999), indicating that a tight regulation of Tctp expression levels is necessary in order for cells to maintain normal growth programs. Apart from being implicated in cellular growth, Tctp plays a role in DNA damage (Zhang et al., 2012) and allergy responses (MacDonald et al., 1995), among other functions (Amson et al., 2013), and has remained highly conserved throughout phylogeny (Brioudes et al., 2010). Its role is particularly well studied in cancer, where high-Tctp expression status is a signature marker of aggressive and advanced breast cancer and glioma, respectively (Amson et al., 2012; Miao et al., 2013). Also of note, decreasing Tctp expression potentiates tumor reversion, a biological mechanism whereby tumorigenic cells lose their malignant phenotype (Tuynder et al., 2002).

Our interest in Tctp arose from the identification of its mRNA amongst the most enriched axonal transcripts in various embryonic and adult neuronal populations (Taylor et al., 2009; Andreassi et al., 2010; Zivraj et al., 2010; Gumy et al., 2011), suggestive of a possible specific role for axonal Tctp. While its importance during axon development processes has been established (Roque et al., 2016), it remains unexplored whether Tctp protein levels are regulated subcellularly in response to guidance signals and to what extent this local modulation contributes to normal axon growth and pathfinding.

*tctp* transcripts contain two clues that are indicative of their mode of regulation: first, the 5′-end of *tctp* mRNAs starts with a canonical 5′-terminal oligopyrimidine (TOP) motif (Thiele et al., 1998; Fiucci et al., 2003; Roque et al., 2016), a typical feature of transcripts selectively regulated at the translational level by mTORC1 (Saxton and Sabatini, 2017). Second, the *tctp* gene is transcribed into two mRNAs that vary only in 3′untranslated region (3′UTR) length as a result of the differential usage of two alternative polyadenylation signals (Thiele et al., 2000; Roque et al., 2016). Although both isoforms are ubiquitously distributed, expression of *tctp* variants differs considerably in quantity and proportion between tissues (Thiele et al., 2000). Intriguingly, both isoforms are abundantly expressed in axons (Roque et al., 2016), suggesting possibly specificity in function.

By reacting to inputs from energy, nutrient, oxygen and growth factor signals, mTORC1 acts as a global rheostat to control protein catabolic and anabolic processes, and thus TOP mRNA translation is intimately associated with overall cellular growth conditions (Saxton and Sabatini, 2017). Accordingly, serum stimulation is known to increase the expression of Tctp in a PI3-K/Akt/mTORC1-dependent manner (Bommer et al., 2015), and its synthesis is greatly boosted in growing vs. non-growing Ehrlich ascites tumor cells (Benndorf et al., 1988; Böhm et al., 1989). Given that pathfinding growth cones alternate between permissive and restrictive environments, many of which act through the mTOR pathway (Campbell and Holt, 2001; Piper et al., 2006; Nie et al., 2010), it is conceivable that guidance cues shape Tctp axonal function via local modulations of its expression and that *tctp* mRNA isoforms add an additional regulatory layer to this process. Furthermore, the enrichment of *tctp* mRNAs in embryonic axons suggests that axonally produced Tctp could contribute to axon development.

Here, we tested whether axonal Tctp expression levels respond to two mTORC1-impacting guidance cues that function in retinal axon guidance: Netrin-1, a cue important during early guidance and branching decisions, and Ephrin-A1, a synaptogenic signal present at termination zones. We report that Tctp expression in growth cones is rapidly and oppositely regulated by these signals and provide evidence in support of local translation being necessary for the upregulation of Tctp in growth cones treated with Netrin-1. Live imaging with translation reporters further showed that Netrin-1-induced synthesis of Tctp in growth cones is driven by a specific mRNA isoform. In spite of this, our investigation also revealed that acute inhibition of *de novo* Tctp translation in axons does not perturb the advance of retinal ganglion cell (RGC) axons through the optic tract.

## Materials and Methods

### *Xenopus laevis* Embryos Maintenance

*Xenopus laevis* embryos were obtained by *in vitro* fertilization, raised in 0.1× Modified Barth’s Saline (0.88 mM NaCl, 0.01 mM KCl, 0.024 mM NaHCO_3_, 0.1 mM HEPES, 8.2 μM MgSO_4_, 3.3 μM Ca(NO_3_)_2_, 4.1 μM CaCl_2_) at 14–18°C and staged according to Nieuwkoop and Faber (1994). This research has been regulated under the Animals (Scientific Procedures) Act 1986 Amendment Regulations 2012 following ethical review by the University of Cambridge Animal Welfare and Ethical Review Body (AWERB).

### Retinal Cultures

Eye primordia were dissected from anesthetized stage 32 embryos, rinsed and then plated on culture dishes pre-coated with poly-L-lysine (10 μg/mL, Sigma) and fibronectin (10 μg/mL, Sigma; Netrin-1 stimulation experiments) or laminin (10 μg/mL, Sigma; Ephrin-A1 stimulation experiments). Cultures were generally incubated at 20°C in serum-free 60% L15 minimal medium (Life Technologies) for 24 h before experimentation, except during experiments with Kaede translation reporters (described below).

### Netrin-1 Stimulation

After 24 h incubation, explants were bathed in 600 ng/mL recombinant mouse Netrin-1 (reconstituted in PBS containing 0.1% (wt/vol) bovine serum albumin) for 5 min and fixed in 2% (vol/vol) paraformaldehyde/7.5% (wt/vol) sucrose; BSA (0.1%) was used as vehicle control. Cycloheximide (CHX; 25 μM, Sigma) and rapamycin (10 nM, Sigma) were bath-applied to cultures 2 min before the addition of Netrin-1. Cultures were subsequently processed for quantitative immunofluorescence analysis. Experimental conditions used when evaluating *tctp* translation dynamics with Kaede reporters are described in detail in the “Kaede-*tctp* Translation Reporters in Cultured Axons” subsection.

### Ephrin-A1 Stimulation

As previously described by Sahin and colleagues (Nie et al., 2010), to pre-aggregate proteins, recombinant mouse Ephrin-A1-Fc (a recombinant chimera between mouse Ephrin-A1 and human IgG_1_ Fc region; R&D Systems) or human IgG_1_ Fc peptide (supplied by Jackson Immunoresearch Labs) were incubated with a goat antibody against the human Fc region (Jackson Immunoresearch Labs). Specifically, anti-human IgG, Fc fragment specific antibody (100 μL, diluted at 0.9 mg/mL) was incubated with Ephrin-A1-Fc (100 μL of 100 μg/mL stock) or 100 μL of IgG_1_ Fc peptide (100 μL, diluted at 100 μg/mL). Following gentle agitation for 45 min at room temperature, protein aggregates were bath-applied to retinal cultures for 10 min (unless otherwise specified) at a 1:10 dilution (final concentration: 5 μg/mL) before fixation. Cultures were subsequently processed for quantitative immunofluorescence analysis.

### Immunostaining and Quantitative Immunofluorescence Microscopy

As described previously (Leung et al., 2013), cells were fixed in 2% (vol/vol) paraformaldehyde/7.5% (wt/vol) sucrose for 20 min, washed multiple times in 1× PBS and permeabilized for 3 min using saponin (1 mg/mL in PBS). Standard immunocytochemistry protocols were followed henceforth (blocking solution: 5% heat-inactivated goat serum in 1× PBS and overnight incubation with primary antibodies at 4°C). Except during fixation, a low concentration of saponin (100 μg/mL) was included to reduce the surface tension of all solutions; this was done in order to minimize explant detachment during processing. Fluorescence intensity (mean pixel intensity per unit area) was measured in non-collapsed growth cones with Openlab (PerkinElmer) or Volocity (PerkinElmer) software. Measurements were taken using masks obtained by manually tracing growth cones in corresponding bright-field images. Background fluorescence was then subtracted from total growth cone fluorescence and data were normalized to the mean immunofluorescence intensity of the control group and expressed as percentage. Our analysis was restricted to growth cone lamelipodia when quantifying Tctp expression due to its punctate distribution pattern in this structure as opposed to its dense occupation of the growth cone central domain. A Nikon Eclipse TE2000-U inverted microscope was used for all image acquisitions. Antibodies: rabbit anti-Tctp raised against *Xenopus* Tctp (1:500, generously gifted by Jacek Z. Kubiak at the French National Centre for Scientific Research); rabbit anti-phospho_S235/236_-rpS6 (1:500, Upstate); mouse anti-ubiquitinylated protein conjugates, clone FK2 (Millipore). Alexa Fluor secondary antibodies (Life Technologies) were used at 1:1,000.

### Cloning and *in vitro* Transcription of Kaede-S and Kaede-L

Generation of chimeric Kaede-*tctp* constructs made use of TA-cloned RACE PCR fragments obtained during the characterization of *tctp* isoforms and an empty CoralHue pKaede-S1 cloning vector (MBL International). The *tctp* 5′UTR was amplified with the following primer pair: 5′-TCAGCAGAATTCCCTTTTCTCTCCCCACCCTCCG-3′ and 5′-TCGCGTGGATCCGTTGGCGGCCTAAGTGTTGTAATG-3′. Primer design included unique restriction sites in the 5′ end (EcoRI and BamHI sites in forward and reserve primers, respectively). Overall, 3′UTR cloning involved an equivalent strategy. The *tctp-s* 3′UTR was amplified with the following primer pair: 5′-CGCACTGCAGCATTCCGTTTGGTTCTTCCATCTT-3′ and 5′-GGGCCCATATGACAGTGGAGAATCATGGGCTTTAT-3′. The *tctp-l* 3′UTR was amplified with the following primer pair: 5′-CGCACTGCAGCATTCCGTTTGGTTCTTCCATCTT-3′ and 5′-CGCGGCCATATGTTGTTTAATTCTGTCTTTATTCAGGATC-3′. Here, forward primers included a PstI restriction site and reverse primers contained a NdeI site. Finally, a T3 phage polymerase promoter was added immediately upstream of the 5′UTR using the following primer combinations: 5′-AATTAACCCTCACTAAAGGCCTTTTCTCTCCCCACCCTCCG-3′ and 5′-GGGCCCATATGACAGTGGAGAATCATGGGCTTTAT-3′ (forward and reverse primers, respectively, for Kaede-S); 5′-AATTAACCCTCACTAAAGGCCTTTTCTCTCCCCACCCTCCG-3′ and 5′-CGCGGCCATATGTTGTTTAATTCTGTCTTTATTCAGGATC-3′ (forward and reverse primers, respectively, for the Kaede-L construct). Capped Kaede mRNAs were synthesized from linearized plasmids using mMESSAGE mMACHINE T3 Transcription kit (Life Technologies); a poly(A) tail was subsequently added using Poly(A) Tailing Kit (Life Technologies). Before delivery, the resultant RNA was column-purified (RNeasy Mini Kit, Qiagen).

### Blastomere Microinjection

Embryos were injected as previously described (Leung and Holt, 2008). Dorsal blastomere injections were performed at the 4-cell stage using a pressurized microinjector (Picospritzer, General Valve) equipped with glass capillary needles (1.0 mm outer diameter × 0.5 mm internal diameter, Harvard Apparatus). mRNA was delivered at 90 pg/blastomere.

### Kaede-*tctp* Translation Reporters in Cultured Axons

Experiments with Kaede reporters were performed as described previously with minor alterations (Leung et al., 2006; Leung and Holt, 2008). Eye primordia were dissected from anesthetized stage 24 Kaede-positive embryos and cultured for 14–18 h before imaging. Culture dishes were pre-coated with poly-L-lysine (10 μg/mL, Sigma) and fibronectin (10 μg/mL, Sigma). RGC axons were manually severed from cell bodies using a mounted pin holder and monitored for signs of blebbing and fragmentation during stimulation protocols. Before Netrin-1 (600 ng/ml) or control stimulation (0.1% BSA), Kaede green was photoconverted using two 5 s pulses of 405-nm laser illumination. Images were acquired at 5 min intervals using an UltraView VoX spinning disk confocal imaging system (PerkinElmer) on an Olympus IX81 microscope. For data analysis, growth cone outlines were traced in Volocity (PerkinElmer); fluorescence recovery was calculated from background-corrected intensity measurements by normalizing green fluorescence change (*F — F_0_*) as percentage change of fluorescence intensity (*F — F*_0_)/*F*_0_, where *F*_0_ is the fluorescence intensity measured at 0 min and *F* the fluorescence intensity of subsequent time points. Unhealthy axons were discarded from analysis.

### Neuronal Culture and Western Blot Analysis

Primary cortical neurons were prepared from embryonic day 16 rat embryos and grown 8 days *in vitro* at 37°C/5% CO_2_ before treatment with rapamycin (50 nM). Immunoblots were performed using antibodies against Tctp (1:500; Santa Cruz Biotechnology, sc-133131), S6 ribosomal protein (1:1,000; Cell Signaling, #2317), phospho_Ser235/236_-S6 Ribosomal Protein (1:1,000; Cell Signaling, #2211), β-Actin (1:5,000; Cell Signaling, #4967) and β-3 Tubulin (1:10,000; Thermo, MA1–118), in combination with HRP-conjugated secondary antibodies from Thermo. Signal development was achieved using 1-Shot Digital ECL reagents (Kindle Biosciences).

### Electroporation and *in vivo* Imaging

Target eye electroporation was performed using methodology previously described (Falk et al., 2007). Stage 28 tadpoles anesthesia was achieved by immersion in 0.4 mg/mL MS222 (tricaine mesylate) in 1× MBS. The retinal primordium was injected with 1 μg/μL pCS2 + mRFP followed by a total of eight 18 V electric pulses of 50 ms duration delivered at 1-s intervals. Following electroporation, embryos were recovered in 0.1× MBS until stage 37/38. At this point, the lateral surface of the brain hemisphere contralateral to the RFP-labeled eye was carefully exposed by removing skin and eye (the removal of the latter was necessary in order to permit unobstructed pathway inspection; Chien et al., 1993). A second round of electroporation followed (eight 18 V/50 ms electric pulses delivered at 1-s intervals) immediately after the delivery of fluorescein-tagged *tctp*- or control morpholinos in the optic pathway vicinity. Electroporation cycle was repeated once to guarantee effective morpholino delivery, as described previously (Wong et al., 2017). The electroporated eye was subsequently removed to eliminate material contribution from the soma. Finally, embryos were transferred to an oxygenated chamber made with Permanox slides (Sigma-Aldrich) and adhesive Gene Frames (Thermo Scientific) containing 1× MBS supplemented with 0.1 mg/mL MS222. Image acquisition was performed using a Nikon Eclipse TE2000-U inverted microscope (objective: Plan Fluor 20× (NA 0.5)) and Volocity software (PerkinElmer).

### Statistical Analysis

Each experiment was repeated at least three times, unless otherwise stated. Details of statistical analysis for each experiment are provided in figure legends. Data were analyzed in Prism 5 (GraphPad). For all experiments, a statistical significance threshold of *α* = 0.05 was used.

## Results

### Tctp Is Locally Synthesized in RGC Growth Cones in Response to Netrin-1

**Figure 1:**
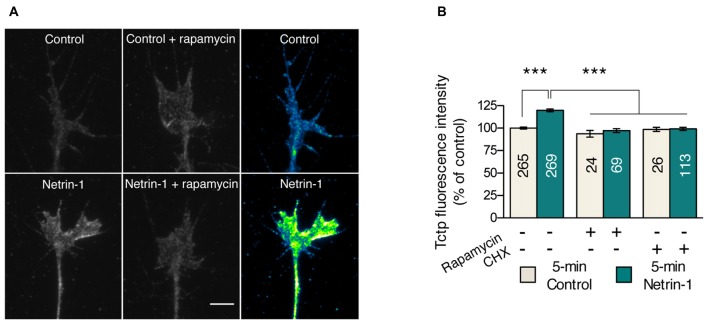
Netrin-1 triggers a translation-dependent rise in growth cone translationally controlled tumor protein (Tctp). **(A,B)** Retinal explants were stimulated with Netrin-1 for 5 min and stained for Tctp. Netrin-1 induced an increase in growth cone Tctp mean intensity signal relative to control, and this effect was prevented by rapamycin or cycloheximide (CHX) pre-treatment (mean ± SEM; *n* = no. of growth cones analyzed; ***P < 0.0001, Kruskal-Wallis test). Scale bars: 5 μm.

We first investigated whether Tctp growth cone levels are regulated by local translation mechanisms. We focused on Netrin-1 because the chemotropic response elicited by this guidance cue requires mTORC1-dependent new protein production (Campbell and Holt, 2001). Knowing *tctp* to be a TOP mRNA, we reasoned Netrin-1 might specifically regulate *tctp* translation in embryonic growth cones. To test this, we performed 5 min Netrin-1 stimulations in cultured *Xenopus* eye explants, a protocol sufficient to induce measurable increases in local protein synthesis (Leung et al., 2006; Cagnetta et al., 2018), and used quantitative immunofluorescence to monitor growth cone Tctp levels. This approach revealed a ~20% increase in the average pixel intensity per unit area in Netrin-1-treated growth cones compared to controls (Figures 1A,B). We next asked whether interfering pharmacologically with the protein translation machinery blocked the observed upregulation of growth cone Tctp expression triggered by Netrin-1. Both CHX, an inhibitor of translation elongation, and rapamycin, an allosteric mTORC inhibitor, prevented the increase in Tctp signal when applied acutely just before the Netrin-1 stimulus (Figures 1A,B and Supplementary Figure S1A). Collectively, these results reveal that Tctp levels in the growth cone are rapidly regulated by Netrin-1, and that this is a protein synthesis- and mTORC1-dependent event.

### Netrin-1-Triggered Tctp Local Translation Is mRNA Isoform-Specific

A body of work has linked the translation of mRNAs to the regulatory effects exerted by the UTRs (Gebauer et al., 2012). We have previously reported that *tctp* expression in *Xenopus laevis*, similar to its human counterpart, is regulated by alternative polyadenylation mechanisms, producing two mRNA isoforms that differ only in the length of their 3′UTR (*tctp-s* and *tctp-l*). Albeit at different levels, the two isoforms are amply expressed in the axonal compartment of embryonic RGC (Roque et al., 2016), indicating that they may both function locally during development and could potentially be regulated by different inputs.

**Figure 2:**
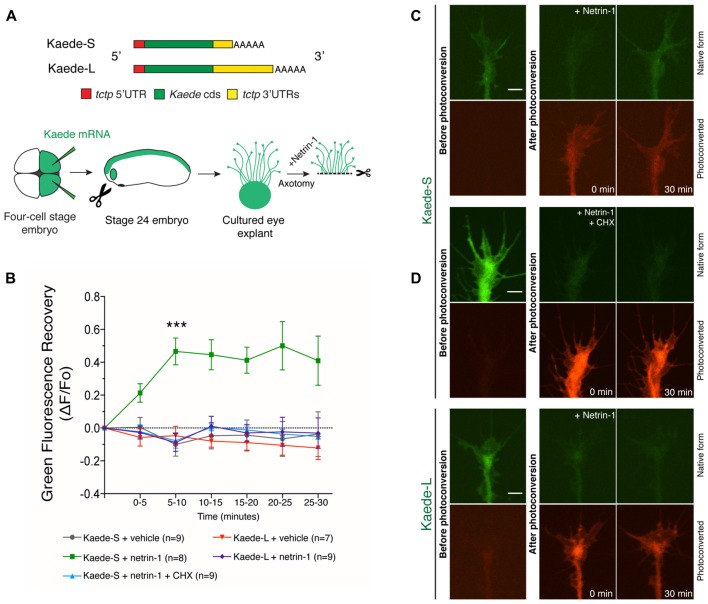
Netrin-1-induced translation reporter synthesis reveals 3′untranslated region (3′UTR) isoform dependancy. **(A)** Schematic of Kaede constructs and experimental design. *In vitro* transcribed mRNAs encoding Kaede-S or Kaede-L were introduced by blastomere injection and stage 24 eye explants cultured for 14–18 h before imaging. Kaede-positive axons were severed from their cell bodies before green-to-red UV-photoconversion and continually examined for signs of cell death (e.g., fragmentation along the axon shaft) thereafter. **(B)** Quantification of green fluorescence recovery over multiple time-lapse sequences (mean ± SEM; *n* = no. of growth cones analyzed; ***P < 0.0001, two-way ANOVA and Bonferroni). **(C,D)** Pre- and post-photoconversion images of severed axons expressing Kaede-S or Kaede-L. Fluorescence recovery in response to Netrin-1 was only observed in Kaede-S-expressing neurons, and this effect was prevented by CHX treatment. Scale bars: 5 μm.

To explore the extent to which the two *tctp* isoforms contribute to the Netrin-1-induced increase in Tctp growth cone levels, we generated Kaede translation reporters flanked by the UTRs of both *tctp* variants (henceforth termed *Kaede-S* and *Kaede-L*; Figure 2A). Kaede protein in its original form emits green fluorescence, but its emission can be irreversibly converted to red by UV illumination (Ando et al., 2002). This spectral transition can be harnessed for the study of new protein translation because it allows for the distinction of new and preexisting protein (Leung et al., 2006). For the purpose of our experiment, *in vitro* transcribed *Kaede-S* or *Kaede-L* mRNAs were introduced by blastomere injection and stage 24 eye explants cultured for 14–18 h before experimentation. Subsequently, Kaede-positive axons were severed from their cell bodies before green-to-red UV-photoconversion and continually examined for signs of cell death (e.g., blebbing and fragmentation along the axon shaft) thereafter (Figure 2A).

Unstimulated, vehicle-treated Kaede-S- or Kaede-L-positive growth cones showed no green fluorescence recovery over the 30 min course of these experiments (Figure 2B; Supplementary Figures S1B,C). By contrast, in Kaede-S-expressing neurons, Netrin-1 application significantly increased the green, but not the red, signal within 10 min of stimulation (Figures 2B,C). Notably, the Netrin-1-induced return of green fluorescence was blocked by CHX added prior to photoconversion, denoting the synthesis of new protein instead of mere fluorophore maturation of previously synthesized Kaede protein (Figures 2B,C). Furthermore, as this elevation in Kaede-green signal occurred in axons severed from their cell bodies, we excluded soma-derived protein as a possible source of new protein. By contrast, even upon stimulation with Netrin-1, no resurgence of green signal in neurons expressing Kaede-L was detected during the 30 min window of these experiments (Figures 2B,D, Supplementary Figure S1D). These observations are not due to an intrinsic inability of the *Kaede-L* mRNA to be translated, as profuse signal was observed before photoconversion (Figure 2D, Supplementary Figure S1C). Collectively, these data indicate that the 3′UTR of *tctp-s* can direct the local translation of new Tctp protein in response to Netrin-1. Also, the findings are in agreement with the possibility that the *tctp-s* isoform is the sole origin of the newly synthesized axonal Tctp protein induced by Netrin-1.

### Ephrin-A1 Repression of mTORC Signaling Is Dependent on Retinal Origin

Growth arrest under a variety of conditions is known to result in the selective repression of 5′-TOP mRNA translation (Meyuhas, 2000; Nandagopal and Roux, 2015). A developing axon may be understood to experience an analogous effect upon reaching its synaptic target area, as numerous cues signal for it to halt its extension program and arborize (Waites et al., 2005). The graded distribution of Eph receptor tyrosine kinases and their ligands, the ephrins, constitute an effective and well-characterized mode of establishing the correct termination zone coordinates for RGC axons (McLaughlin and O’Leary, 2005; Cang and Feldheim, 2013). As EphA receptor activation by Ephrin-A1 has been shown to dampen mTORC1 activity and protein synthesis in RGC axons via ERK1/2-Tsc2-Rheb signaling (Nie et al., 2010), we considered the possibility that growth cone contact with Ephrin-A1 influences local Tctp expression.

**Figure 3:**
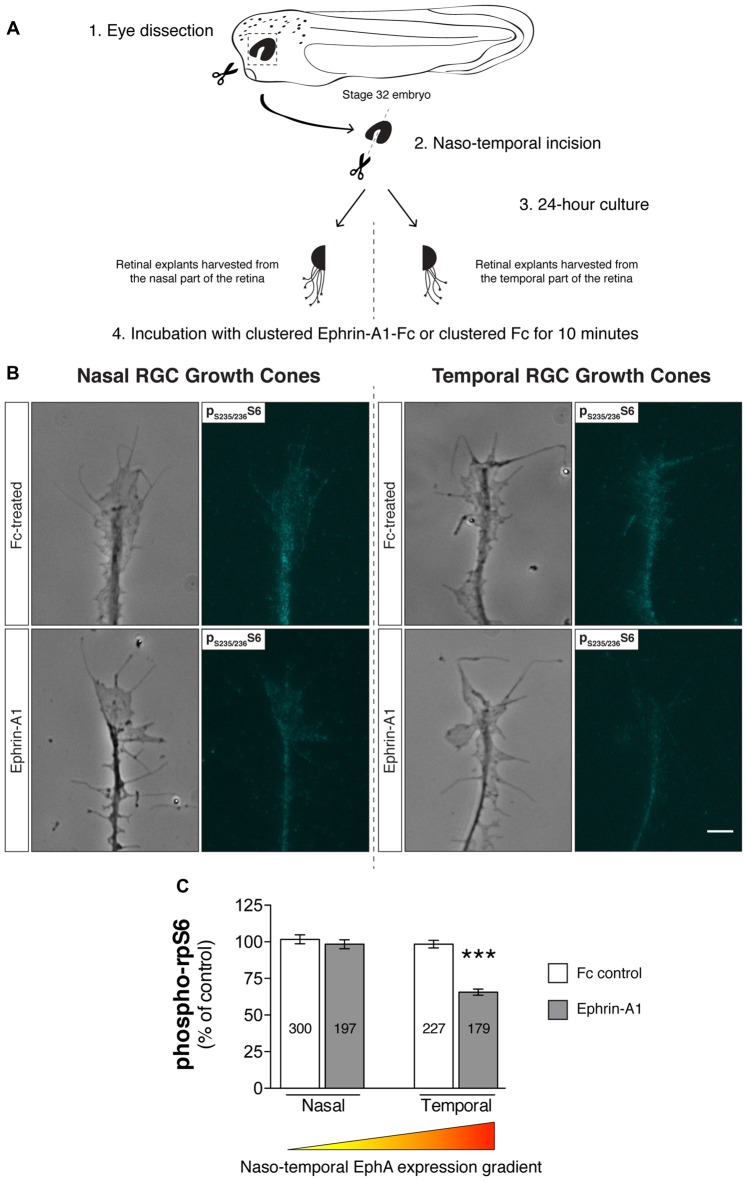
Retinal ganglion cell (RGC) origin dictates distinct growth cone mTOR activation profiles in response to Ephrin-A1. **(A–C)** Nasal and temporal stage 32 retinal explants grown *in vitro* for 24 h were stimulated with Ephrin-A1-Fc at a concentration of 5 μg/mL, and stained with an antibody that specifically recognizes ribosomal protein S6 (rpS6) when phosphorylated at Ser-235/236. Representative micrographs of control (clustered Fc)- and Ephrin-A1-treated RGC growth cones are shown (mean ± SEM; *n* = no. of growth cones analyzed; ***P < 0.0001, one-way ANOVA). Scale bar: 5 μm.

Aiming to corroborate the findings by Nie et al. (2010) in our model system and simultaneously gain insight into this signaling mechanism, we first asked if the EphA receptor gradient that exists across the retinal nasal-temporal axis affects the inhibitory effects on mTORC1 signaling elicited by Ephrin-A1. To this end, explants from temporal and nasal retinal crescents of stage 32 *Xenopus* embryos were cultured for 24 h (Figure 3A), a duration that approximately equates *in vitro* to the arrival of RGC axons in the Ephrin-A-expressing optic tectum *in vivo* (Zivraj et al., 2010). Notably, following a 10 min incubation protocol with Ephrin-A1 (5 μg/mL), we observed using quantitative immunofluorescence a significant decrease (approximately 35%; *P* < 0.0001, one-way ANOVA and Tukey’s Multiple Comparison Test) in ribosomal protein S6 (rpS6) phosphorylation levels at Ser_235/236_, a cardinal marker of mTORC1 activity (Biever et al., 2015), in temporal RGC growth cones (Figures 3B,C). In contrast, no detectable effects were detected in RGC growth cones originating from nasal crescents (Figures 3B,C). Overall, these findings validate and expand on previous observations, suggesting that the level of expression of EphAs receptors is a fundamental variable in the outcome of Ephrin-A1-induced inhibition of mTORC1.

### Ephrin-A1 Downregulates Growth Cone Tctp Levels

**Figure 4:**
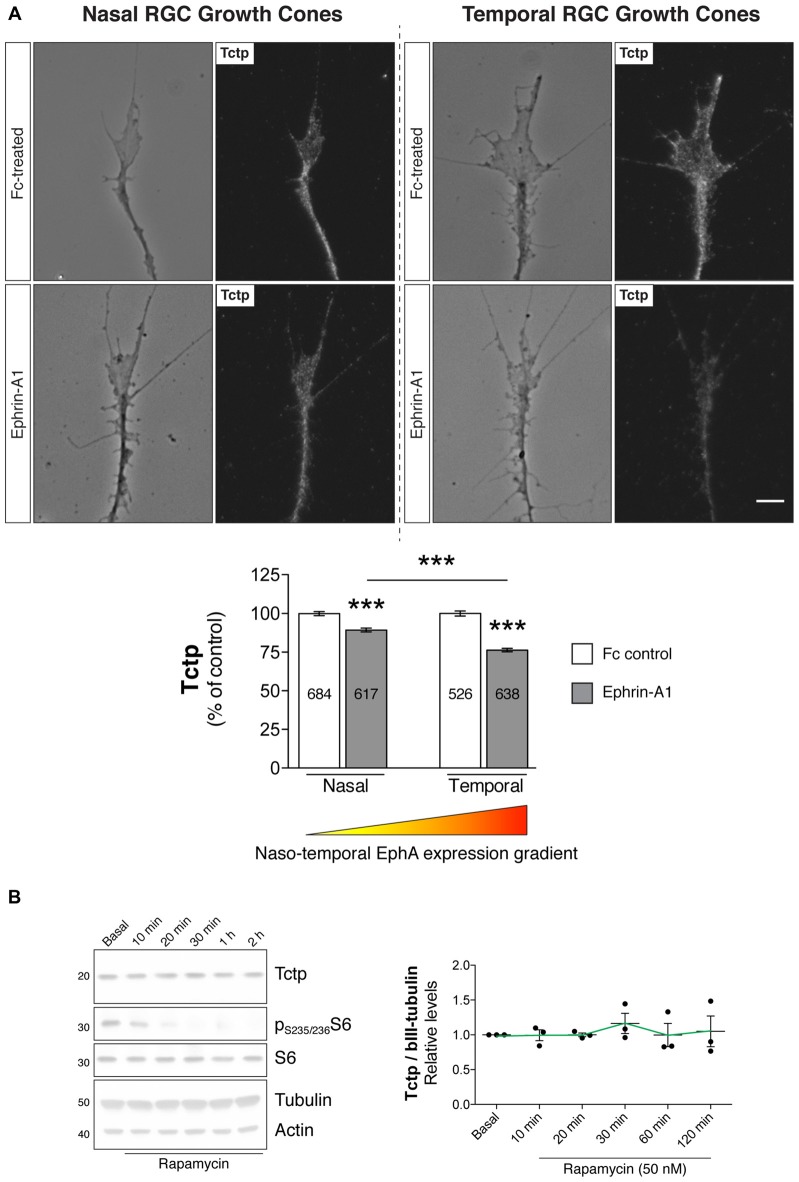
Ephrin-A1 reduces growth cone Tctp expression levels. **(A)** Nasal and temporal stage 32 retinal explants grown *in vitro* for 24 h were stimulated with Ephrin-A1-Fc at a concentration of 5 μg/mL, and stained for Tctp. Representative micrographs of control (clustered Fc)- and Ephrin-A1-treated RGC growth cones are shown (mean ± SEM; *n* = no. of growth cones analyzed; ***P < 0.0001, one-way ANOVA and Tukey’s Multiple Comparison Test). **(B)** Rat primary cortical neurons were incubated with rapamycin at 50 nM for up to 2 h and Tctp protein levels evaluated in whole cell extracts at various time points (mean ± SEM; *n* = 3 biological replicates; *P* = 0.9282, one-way ANOVA). Scale bar: 5 μm.

Having confirmed that EphrinA1-EphA (forward) signaling modulates the activation of the mTORC pathway in *Xenopus* RGCs, we next examined whether EphrinA1-EphA-mediated signaling also regulated the expression of growth-promoting Tctp in the growth cone. Employing the same stimulatory protocol as before (Figure 3A), we observed an inverse correlation between EphA activation and Tctp expression using quantitative immunofluorescence: Tctp protein levels in temporal growth cones showed a reduction of approximately 25% relative to temporal controls (*P* < 0.0001, one-way ANOVA and Tukey’s Multiple Comparison Test), accompanied by a smaller decline in growth cone Tctp levels in nasal counterparts (circa 10% vs. nasal controls; *P* < 0.0001, one-way ANOVA and Tukey’s Multiple Comparison Test; Figure 4A). Collectively, these results indicate that, at least to some extent, the levels of Tctp reflect the activation of mTORC1 in Ephrin-A1-treated growth cones. The observation that nasally derived growth cones, which are unaffected by Ephrin-A1 stimulation in respect to mTORC1 activation (Figures 3B,C), also showed decreased levels of Tctp indicates, however, that additional regulatory mechanisms are in operation in this signaling context. In line with this idea, Tctp protein expression remains surprisingly constant for up to 2 h in neurons treated with rapamycin, even though phospho-S6 levels drop at a rapid pace as soon as 10 min into the treatment (Figure 4B). With this in mind, we sought to explore whether the proteasome might be activated in growth cones in contact with Ephrin-A1, as has been documented for B-type Eph-Ephrin signaling (Mann et al., 2003). Using an antibody that marks mono- and polyubiquitinated protein conjugates (but not free ubiquitin; clone FK2), we saw that growth cones originating from nasal and temporal retinal crescents exhibited an equal overall effect: a reduction in total immunofluorescence signal Supplementary Figure S3). Whereas this result alone does not distinguish between a rapid turnover of protein by the ubiquitin-proteasome system (UPS) or a general increase in deubiquitinase activity, it indicates the involvement of this complex signaling system in Ephrin-A1-mediated responses.

### Axonally Synthesized Tctp Is Not Required for Short-Term Axon Extension *in vivo*

**Figure 5:**
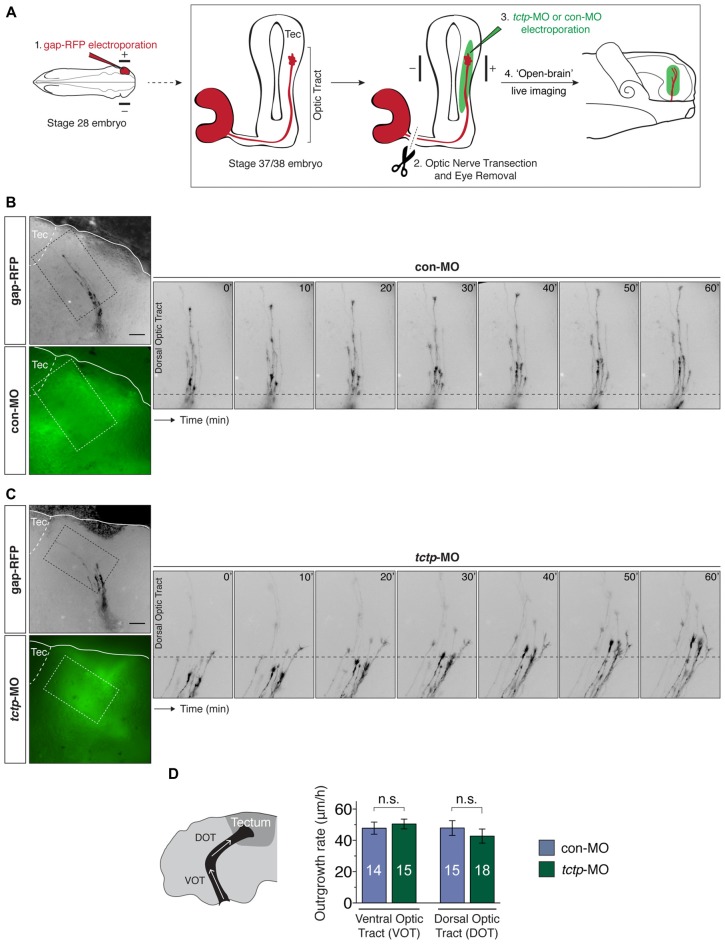
Local synthesis of Tctp is not required for axon extension *in vivo*. **(A)** Gap-RFP was delivered by eye-targeted electroporation to wild-type stage 28 embryos, when the first RGC axons have just exited the eye, and axon extension kinetics were analyzed 21–24 h later (stage 37/38), when most axons are growing up the optic tract towards the optic tectum. Just before imaging, the optic nerve was manually transected, and control morpholino oligonucleotide (con-MO) or *tctp*-MO was delivered subcellularly by targeted brain electroporation. **(B,C)** Time-lapse series of severed RGC axons coursing through the optic tract in embryos subcellularly electroporated with con- or *tctp*-MO. **(D)** Quantication of axon outgrowth rates through the VOT and DOT in embryos subcellularly electroporated with con- or *tctp*-MO (mean ± SEM; *n* = no. of axons analyzed; VOT: *P* = 0.5956, unpaired *t*-test; DOT: *P* = 0.4381, unpaired *t*-test; 7–9 replicates were analyzed per condition; n.s., not significant, unpaired *t*-tests). Scale bars: 50 μm.

We have previously found that Tctp regulates retinal axon growth and guidance programs *in vivo* (Roque et al., 2016). Briefly, Tctp knockdown in the CNS results in slowed and erratic RGC axon extension through the optic tract leading to a delayed innervation of the optic tectum, where RGC axons terminate. Whether this phenotype arises as a consequence of a cell-wide and/or axon-specific disruption in Tctp function remains, however, an open question. In support of the latter, the enrichment of *tctp* messages in embryonic retinal axons suggests that Tctp holds an important local role in developing axons. Prompted by the finding that local protein synthesis regulates growth cone *tctp* expression in response to Netrin-1, we next investigated whether new Tctp production in axons is necessary for the normal development of the *Xenopus* retinotectal projection. To address this question directly *in vivo*, we measured the outgrowth dynamics of transected pathfinding axons advancing through the optic tract in which *tctp* mRNA translation was subcellularly blocked. This was achieved by delivering an antisense morpholino oligonucleotide (MO) into the optic tract/optic tectum region by electroporation to specifically prevent the translation of *tctp* in the axonal compartment only (i.e., without affecting the soma), an approach previously validated by our group (Yoon et al., 2012; Wong et al., 2017). First, a gap-RFP cDNA reporter was electroporated into stage 28 embryonic eyes to enable the detection of live retinal axons in the optic tract; second, *tctp*-MO (which targets both *tctp* isoforms) was electroporated into the optic tract region at stage 37/38 when the pioneer population of retinal axons are growing through the optic tract; third, the eye was surgically removed to eliminate a contribution of new material from the soma; fourth, *in vivo* time-lapse imaging was performed to assess axon growth rate and behavior (Figure 5A). In doing so, we specifically targeted the pool of newly synthesized axonal Tctp and, as transected RGC axons extend and navigate correctly *in vivo* for up to 3 h (Harris et al., 1987), simultaneously eliminated the effects of soma-derived protein without compromising guidance dynamics.

In contrast to global Tctp knockdowns, the axonal delivery of *tctp*-MO did not affect the outgrowth dynamics of RGC axons along the optic tract during the 1 h period of analysis when compared to control embryos (ventral optic tract: 50.5 ± 3.1 μm/h vs. 47.8 ± 3.9 3.1 μm/h in controls; dorsal optic tract: 42.7 ± 4.5 μm/h vs. 47.8 ± 4.7 μm/h in controls; Figures 5B–D). In addition, axon trajectories remained normal in both conditions, even though a major guidance decision—the caudal turn in the mid-optic tract—was made by many axons in the time course of the analysis. These results indicate that the fine, spatiotemporal precise regulation of axonal Tctp expression achieved by local translation mechanisms is not essential, at least acutely, to support the advance of RGC axons through the optic tract.

## Discussion

Different lines of study have established that Tctp cellular levels are regulated in response to intra- and extracellular inputs, including growth signals and certain cytokines (Böhm et al., 1989; Nielsen et al., 1998; Teshima et al., 1998; Bommer et al., 2015), though the biological significance of this adaptive response remains largely unknown. Comparably, in the context of neuronal circuitry formation, we observed protein synthesis-dependent increases in growth cone Tctp expression levels after acute treatment with Netrin-1 and a rapid decline in growth cones exposed to Ephrin-A1, suggesting that Tctp subcellular levels are actively regulated during axon guidance processes. Furthermore, the distinct *Kaede-S* and *Kaede-L* mRNA translation profiles, analogs of the two *tctp* mRNA isoforms, imply that *tctp* transcripts containing the shorter 3′UTR variant (*tctp-s*) may be the sole origin of the *de novo* synthesized Tctp protein observed upon Netrin-1 stimulation. While not excluding that different cues or cellular conditions may activate its translation, we speculate that the longer 3′UTR variant (*tctp-l*) may harbor Netrin-1-independent regulatory motifs. Indeed, *tctp-l* transcripts are predicted to possess many additional stem-loop structures and miRNA-binding sites within their unique 3′UTR stretch, and may therefore associate as part of different ribonucleoprotein particles and/or be subjected to different regulatory constraints (Supplementary Figure S2). Interestingly, *tctp-s* is the predominant form found in embryonic RGC axons, outnumbering *tctp-l*~17-to-1 (Roque et al., 2016). At first glance, one would be inclined to assume *tctp-l* contains a soma-restricting element of some sort. However, the expression level of *tctp-l* in the axonal compartment is almost on par with that of *actb*, a standard axon-enriched mRNA (qPCR analysis showed for every *tpt1-L* message populating the axon, there are two encoding for β-actin; Roque et al., 2016). That is, on these terms, *tctp-l* cannot be ruled out as a weakly expressed isoform in axons; instead, this suggests that the *tctp-l* mRNA variant is also actively trafficked into axons. It is also noteworthy that shorter 3′UTRs are associated with more efficient translation kinetics (Sandberg et al., 2008), a feature that can help explain why a simpler transcript might actually be favored. Still, the clearest hint to the overall cellular significance of *tctp* isoforms is perhaps found in mouse and rat genomes: these species, unlike frog, rabbit and human, do not express the longer 3′UTRs *tctp* variant (Thiele et al., 1998, 2000; Roque et al., 2016). This enigmatic evolutionary history hints that the core functions of Tctp may be regulated solely through *tctp-s*. The reappearance of the longer *tctp* mRNA variant in rabbit and human with relatively little homology to frog* tctp-l* does however suggest that *tctp-l*/Tctp may have acquired new biological roles later in vertebrate evolution (Supplementary Figure S4). Corroborating this possibility, two *TCTP* paralogs have recently been characterized in the parasite *Trypanosoma brucei—*the two genes share identical 5′UTRs and very similar open reading frames, but differ significantly in terms of 3′UTR length, sequence and function: depletion of *TCTP1* is involved in normal cell growth in procyclic-form parasites, whereas when in the bloodstream of a host the parasite depends on *TCTP2* for its growth (Jojic et al., 2018).

While studying how Tctp growth cone expression is modulated by Ephrin-A1, we observed that the state of mTORC activation upon contact with this cue depends on the topographic origin of RGCs in the embryonic retina. Given that the Ephrin-Eph receptor system is a key determinant of retinotectal topographic map formation, this finding potentially offers new mechanistic insight into how Ephrin-A1 gradients coordinate the arrangement of retinal neurons in the brain to reflect that of light-sensitive cells in the retina. Overall, the data are in agreement with the possibility that a progressive inhibition of mTOR signaling, promoted by increasing Ephrin-A1 local concentrations, can play a role in the fine modulation of this response, so that it occurs for each given growth cone only when its termination zone is reached and not before (Supplementary Figure S5). As for Tctp, the observation that its expression levels are rapidly reduced upon contact with Ephrin-A1 indicates that, besides an attenuation of mTORC signaling, additional pathways may be involved in this context. Indeed, while mTORC1 inhibition increases UPS-dependent protein degradation by yet not fully understood mechanisms (Zhao et al., 2015; Rousseau and Bertolotti, 2016; Saxton and Sabatini, 2017), our data argue against mTOR attenuation being solely responsible for the decline in growth cone Tctp levels seen upon Ephrin-A1 stimulation. Notably, the proteasome participates in B-type Eph-Ephrin growth cone responses (Mann et al., 2003), and EphA4 is known to regulate synaptic plasticity through a degradation pathway that requires the ubiquitin proteasome system (Fu et al., 2011). Links between the proteasome, the Rac1 inhibitor chimaerin, and Eph receptor-dependent axon guidance have also been suggested (Hamilton and Zito, 2013). Our own observations indicate that Ephrin-A1 affects protein ubiquitination levels to a similar degree in growth cones derived from nasal and temporal crescents, a somewhat puzzling finding considering that the level of Eph-Ephrin signaling is so different between the nasal and temporal retina. In other contexts, Tctp regulation has been found to be dependent not only on ubiquitination, but also acetylation, phosphorylation, and sumoylation (Vidal, 2017). For example, acetylation controls Tctp degradation by chaperone-mediated autophagy, a proteolytic mechanism that, differently from regular macroautophagy, allows the degradation of specific cytosolic proteins by the lysosome on a molecule-by-molecule fashion (Bonhoure et al., 2017). Based on this, we speculate that the progressive attenuation of mTORC signaling across the nasal-temporal axis coupled with heightened degradative activity triggered by Ephrin-A1 dictate the expression of Tctp in the growth cone. However, considering that the addition/removal of ubiquitin tags affects not only protein degradation by the proteasome and the lysosome, but also shapes protein function and localization, further investigation is needed to pinpoint the nature of growth cone Tctp regulation by Ephrin-A1.

We have found previously that Tctp is required for the development of retinal circuitry by mediating axon growth and guidance (Roque et al., 2016). Specifically, RGC axons in Tctp morphants grow more slowly and stall more frequently in the optic tract segment of the journey towards the optic tectum, their target area in the midbrain. Whether this is due to a cell-wide and/or axon-specific disruption in Tctp function remained, however, unresolved. Here, prompted by the finding that its expression is regulated locally by guidance cues, we examined whether locally produced Tctp plays a role in the normal development of axons advancing through the optic tract. The new analysis that we present indicates that axonal synthesized Tctp is not necessary, at least in the short term, for the progression of RGC axons through the optic tract. This leads us to conclude that the axon phenotypes seen in Tctp morphants likely develop not as consequence of a regionally delimited dysregulation arising as axons advance in the optic tract—local protein synthesis allows for precise spatiotemporal patterns of gene expression—but rather from a prolonged depletion of Tctp levels. While our experiments do not exclude that locally synthesized Tctp can contribute, in the “long run,” to axon guidance processes, its appreciable half-life (*t*_1/2_ > 16 h) may explain why growing axons are able to maintain sufficient Tctp activity when its translation is acutely inhibited (Amzallag et al., 2004; Baylot et al., 2012). It is also possible that locally synthesized Tctp is important only after long-range axon navigation is completed and terminal branching and synaptogenesis have started in the target. In support of this, *in vivo* ribosome immunoprecipitation evidence shows that *tctp* and other TOP mRNAs are locally translated in actively wiring RGC axons, but not before (Shigeoka et al., 2016). This idea is reinforced by a recent report excluding local protein synthesis in distal axons as a requisite for retinal axon growth and navigation, being relevant only during axonal branching (Wong et al., 2017). Irrespective of this, it is enticing to conjecture about an alternative possibility that reflects on the disparity between axonal *tctp* mRNA and protein expression. Intriguingly, although *tctp* transcripts have consistently been placed alongside the most abundant in various whole-genome analyses of embryonic and adult axonal transcriptomes (Taylor et al., 2009; Andreassi et al., 2010; Zivraj et al., 2010; Gumy et al., 2011), proteomic datasets have not corroborated this notion (Estrada-Bernal et al., 2012; Chuang et al., 2018), hinting at a non-coding role for *tctp* mRNAs in axons. As noted in Supplementary Figure S2 and first reported by Clemens and colleagues, *tctp* transcripts are highly structured and have been shown to bind and activate the double-stranded RNA (dsRNA)-dependent protein kinase PKR (Bommer et al., 2002), an intracellular stress sensor upstream of eIF2a, as well as with other physiological roles in signaling (Dabo and Meurs, 2012). Moreover, mammalian genomes have retained a large number of processed *tctp* pseudogenes (Thiele et al., 2000), many even appreciably localizing to neurites (Zappulo et al., 2017). This is relevant considering that pseudogenes can act as competing RNAs (ceRNAs)—i.e., microRNA sponges—and thus it is conceivable that *tctp* and/or its pseudogenes have an important influence as post-transcriptional regulators of gene expression in axons (Tay et al., 2014).

In summary, we have shown that growth cone Tctp expression is dynamically regulated by guidance cues during axon guidance processes. While local protein synthesis contributes to this adaptive response, new Tctp protein production in axons appears to be dispensable for the normal progression of RGC axons through the optic tract. We observed also that RGC origin dictates distinct growth cone mTOR activation profiles in response to Ephrin-A1, a preliminary finding with potential implications to the understanding of visual topographic map formation.

## Author Contributions

CR and CH conceived the project and designed the experiments and wrote the manuscript. CR performed and analyzed the experiments.

## Conflict of Interest Statement

The authors declare that the research was conducted in the absence of any commercial or financial relationships that could be construed as a potential conflict of interest.

## Supplementary Material

FIGURE S1 Netrin-1 triggers a translation-dependent rise in growth cone Tctp. **(A)** Stage 32 retinal growth cones were stimulated with Netrin-1 for 5 min, stained for Tctp and fluorescence intensities were measured. Netrin-1 induced an increase in Tctp mean intensity signal relative to control (Figures 1A,B), which was blocked by pre-incubation with CHX. **(B)** Pre- and post-photoconversion images of severed RGC axon expressing Kaede-S in the presence of 0.1% BSA (vehicle control). **(C)** Pre- and post-photoconversion images of severed RGC axon expressing Kaede-L in the presence of 0.1% BSA (vehicle control). **(D)** Green fluorescence recovery plots of individual time-lapse sequences. Only growth cones stimulated with Netrin-1 are plotted. Scale bar: 5 μm.

FIGURE S2 *tctp* encodes highly structured mRNAs with multiple miRNA binding sites. **(A,B)** *Xenopus laevis tctp-s* and *tctp-l* *in silico* secondary structure prediction using the online RNAfold server. The structures are colored by base-pairing probabilities, with pale colors indicating low base-pairing probabilities. For unpaired regions the color denotes the probability of being unpaired. The unique portion of the *tctp-s* 3^′^UTR is highlighted in light blue. The locations of the main RNA landmarks, such as start and stop codons, are also indicated. **(C)** *In silico*-predicted miRNAs targeting *Homo sapiens tctp* transcripts. The online “miRNA_targets” portal (developed by Amit Kumar and Christophe Lefevre, Deakin University, Australia) used in this analysis employs the miRanda and RNAhybrid algorithms in its predictions.

FIGURE S3 Ephrin-A1-induced decline in growth cone mono- and polyubiquinated protein conjugates is not dependent on topographic origin. Nasal and temporal stage 32 retinal explants grown *in vitro* for 24 h were stimulated with Ephrin-A1-Fc at a concentration of 5 μg/mL for 5 min, and stained with an antibody that specifically recognizes K^29^-, K^48^-, and K^63^-linked mono- and polyubiquinated proteins. Representative micrographs of clustered Fc- and Ephrin-A1-treated retinal ganglion cell growth cones are shown (mean ± SEM; *n* = 2 biological replicates, with 50–100 growth cones analyzed per condition; ****P* < 0.0001; ns, not significant, one-way ANOVA and Tukey’s Multiple Comparison Test).

FIGURE S4 Poor sequence conservation between *Xenopus laevis* and *Homo sapiens tctp-l*. **(A,B)** Sequence alignment of *Xenopus laevis*, *Oryctolagus cuniculus* (rabbit), and *Homo sapiens* *tctp* 3′UTRs using T-Coffee. Both frog (73%) and rabbit (97%) show appreciable sequence homology relative to *Homo sapiens* upstream of the first polyadenylation signal. By contrast, the level of sequence conservation drops significantly in frog downstream of this motif (that is, in region corresponding to the unique stretch of the *tctp-l* 3′UTR), but remains nearly unchanged between rabbit and human. The boxed areas denote the location of the first polyadenylation signal (AATAAA).

FIGURE S5 A model for how increasing Ephrin-A1 local concentrations can lead to a progressive inhibition of mTORC signaling across the naso-temporal axis during topographic map formation. **(A)** RGC axons originating from the temporal retina are high-EphA-expressing and project to the anterior-most, low-Ephrin-A1-expressing regions of the optic tectum. In turn, nasally-derived, low-EphA-expressing RGCs extend past the anterior optic tectum towards posterior (and high-Ephrin-A1-expressing) regions of the target field. This feature makes nasal processes unresponsive to low-Ephrin-A1-territories that temporal axons find restrictive, allowing nasal axons to project to the back of the optic tectum, where higher—and increasingly less permissive—Ephrin-A1 concentrations are found. It follows that low-EphA-expressing nasal RGC growth cones must have a higher threshold of response to Ephrin-A1, otherwise they would terminate precociously along the anterior-posterior axis. In other words, once inside the optic tectum, the relative progression of a RGC growth cone is dependent on the level of EphA receptor expressed on its surface, precisely matched to a repellent Ephrin-A1 counter-gradient—it is this molecular complement that defines the anterior-posterior coordinates of each discrete termination zone (McLaughlin and O’Leary, 2005). **(B)** Our findings suggest that a progressive inhibition of mTORC signaling promoted by increasing Ephrin-A1 local concentrations can potentially play a role in the fine modulation of this response, so that it occurs for each given growth cone only when its termination zone is reached and not before. TN, temporal-nasal axis; AP, anterior-posterior axis.
