# Supplementary figures and images for: Growth Cone Tctp Is Dynamically Regulated by Guidance Cues
Source: Front Mol Neurosci. 2018 Nov 6;11:399. doi: 10.3389/fnmol.2018.00399 (PMC6232380; doi:10.3389/fnmol.2018.00399)

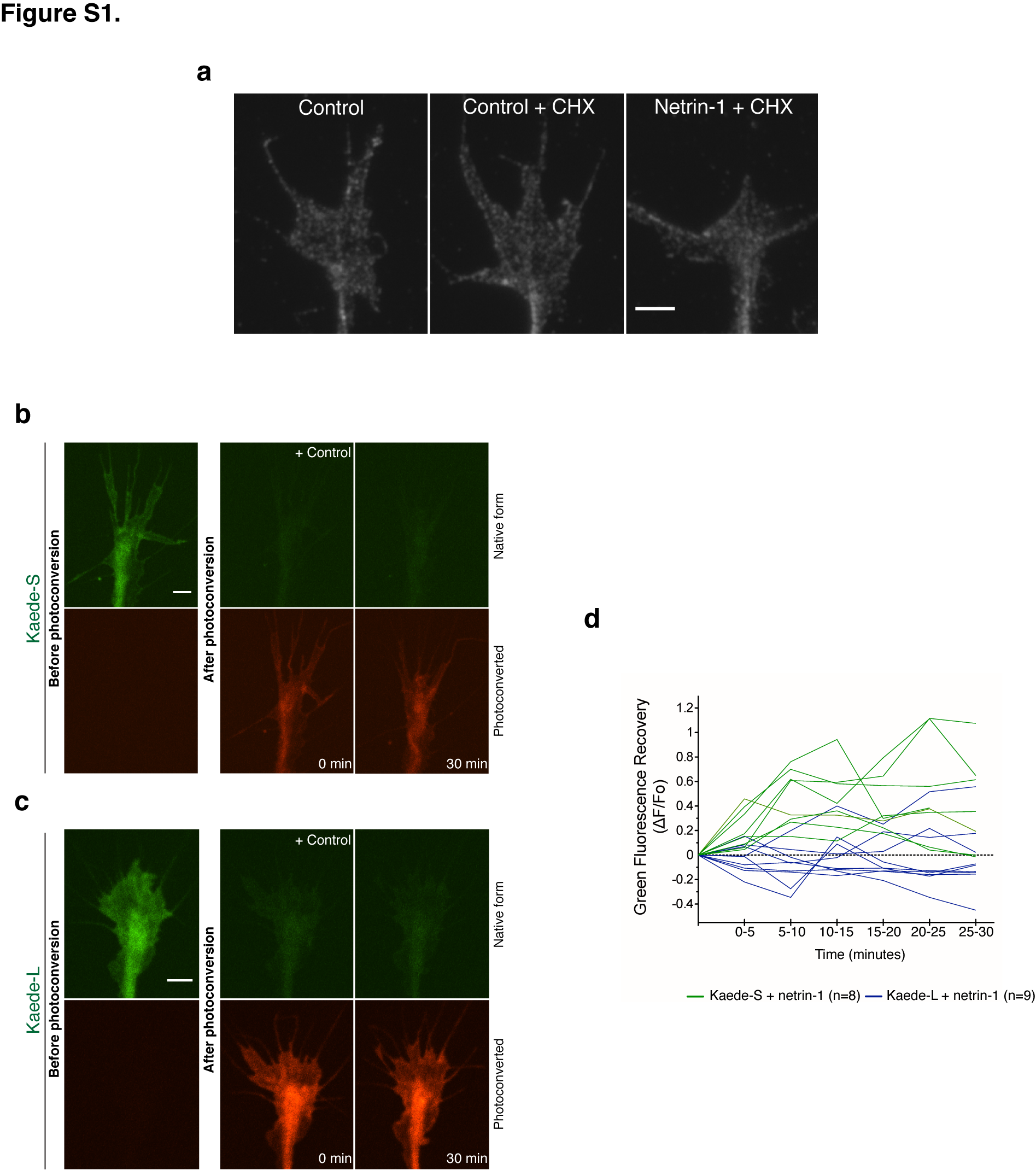

Supplement: FIGURE S1 — Netrin-1 triggers a translation-dependent rise in growth cone Tctp. (A) Stage 32 retinal growth cones were stimulated with Netrin-1 for 5 min, stained for Tctp and fluorescence intensities were measured. Netrin-1 induced an increase in Tctp mean intensity signal relative to control (Figures 1A,B), which was blocked by pre-incubation with CHX. (B) Pre- and post-photoconversion images of severed RGC axon expressing Kaede-S in the presence of 0.1% BSA (vehicle control). (C) Pre- and post-photoconversion images of severed RGC axon expressing Kaede-L in the presence of 0.1% BSA (vehicle control). (D) Green fluorescence recovery plots of individual time-lapse sequences. Only growth cones stimulated with Netrin-1 are plotted. Scale bar: 5 μm. [file Image_1.TIF]

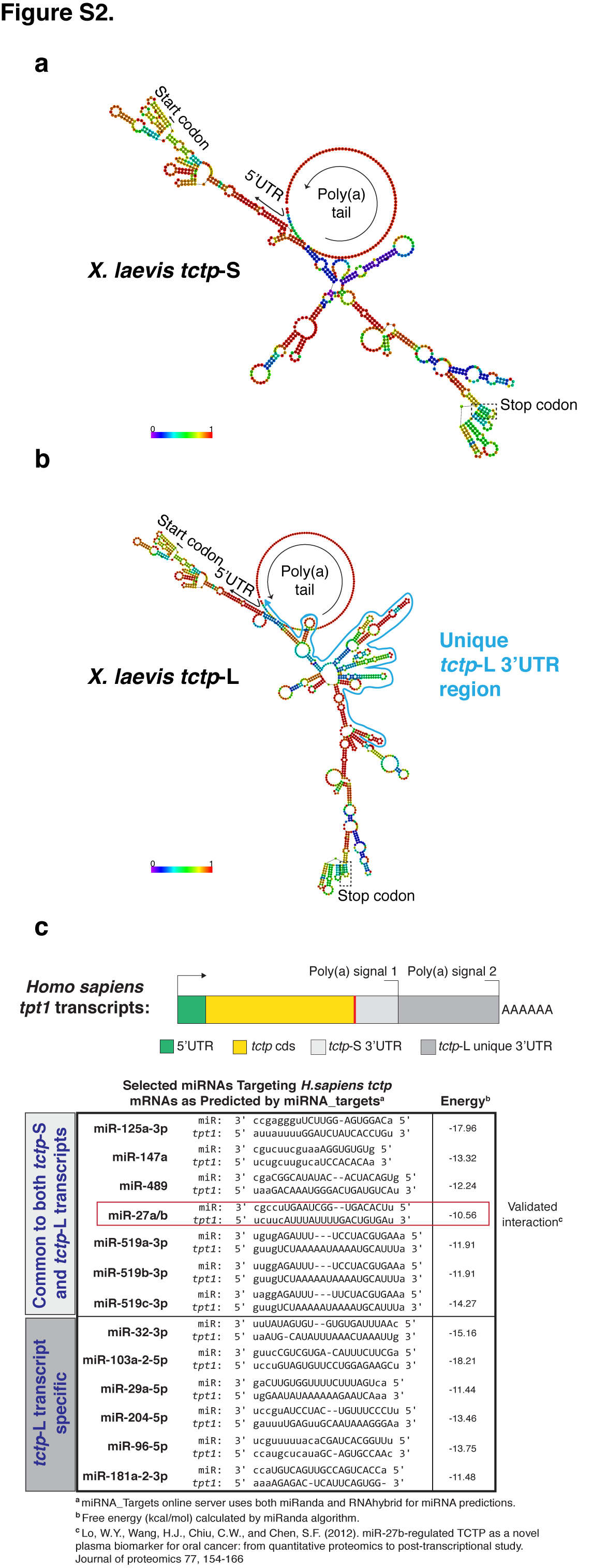

Supplement: FIGURE S2 — tctp encodes highly structured mRNAs with multiple miRNA binding sites. (A,B) Xenopus laevis tctp-s and tctp-l in silico secondary structure prediction using the online RNAfold server. The structures are colored by base-pairing probabilities, with pale colors indicating low base-pairing probabilities. For unpaired regions the color denotes the probability of being unpaired. The unique portion of the tctp-s 3′UTR is highlighted in light blue. The locations of the main RNA landmarks, such as start and stop codons, are also indicated. (C) In silico-predicted miRNAs targeting Homo sapiens tctp transcripts. The online “miRNA_targets” portal (developed by Amit Kumar and Christophe Lefevre, Deakin University, Australia) used in this analysis employs the miRanda and RNAhybrid algorithms in its predictions. [file Image_2.TIF]

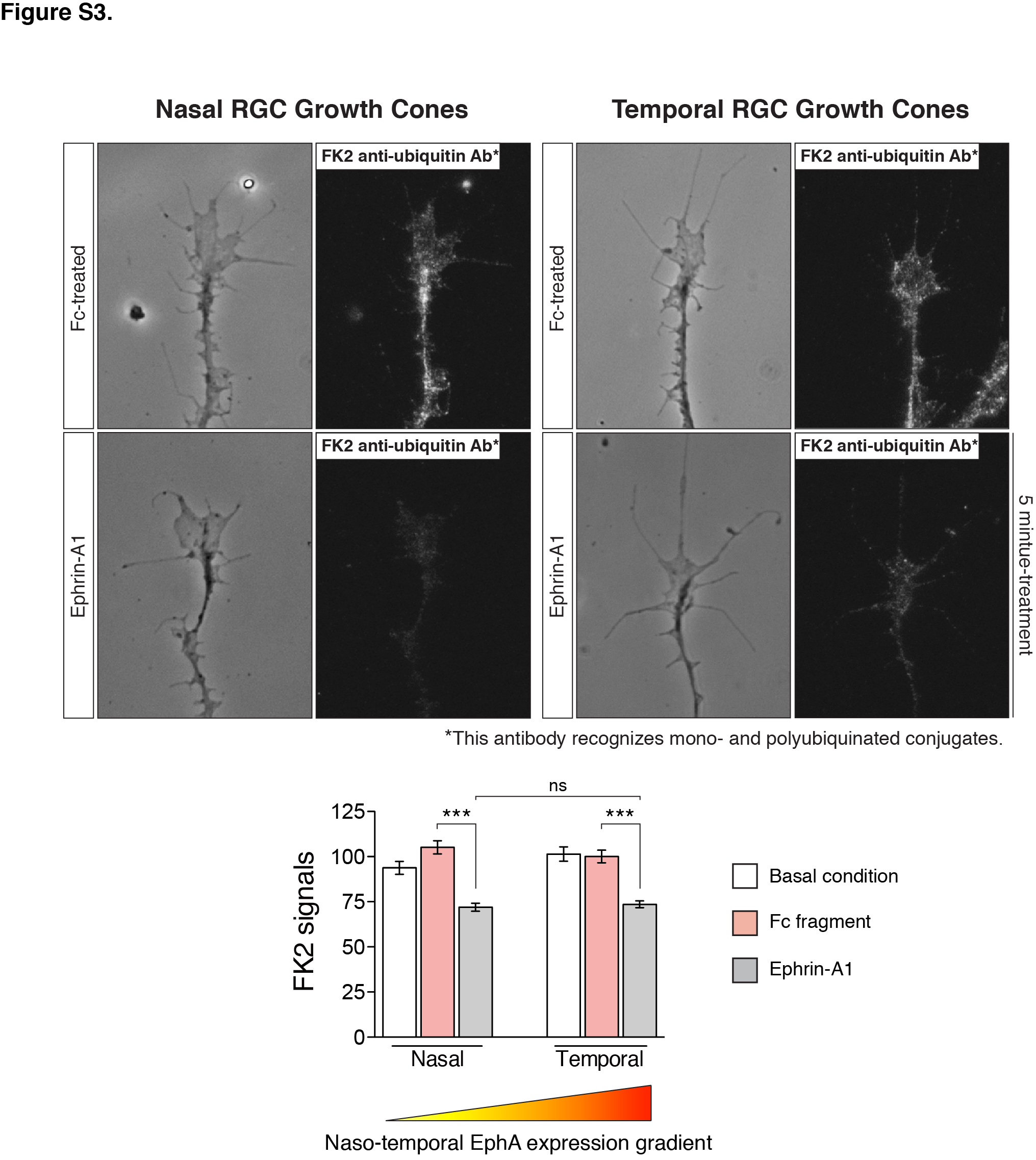

Supplement: FIGURE S3 — Ephrin-A1-induced decline in growth cone mono- and polyubiquinated protein conjugates is not dependent on topographic origin. Nasal and temporal stage 32 retinal explants grown in vitro for 24 h were stimulated with Ephrin-A1-Fc at a concentration of 5 μg/mL for 5 min, and stained with an antibody that specifically recognizes K29-, K48-, and K63-linked mono- and polyubiquinated proteins. Representative micrographs of clustered Fc- and Ephrin-A1-treated retinal ganglion cell growth cones are shown (mean ± SEM; n = 2 biological replicates, with 50–100 growth cones analyzed per condition; ***P < 0.0001; ns, not significant, one-way ANOVA and Tukey’s Multiple Comparison Test). [file Image_3.jpg]

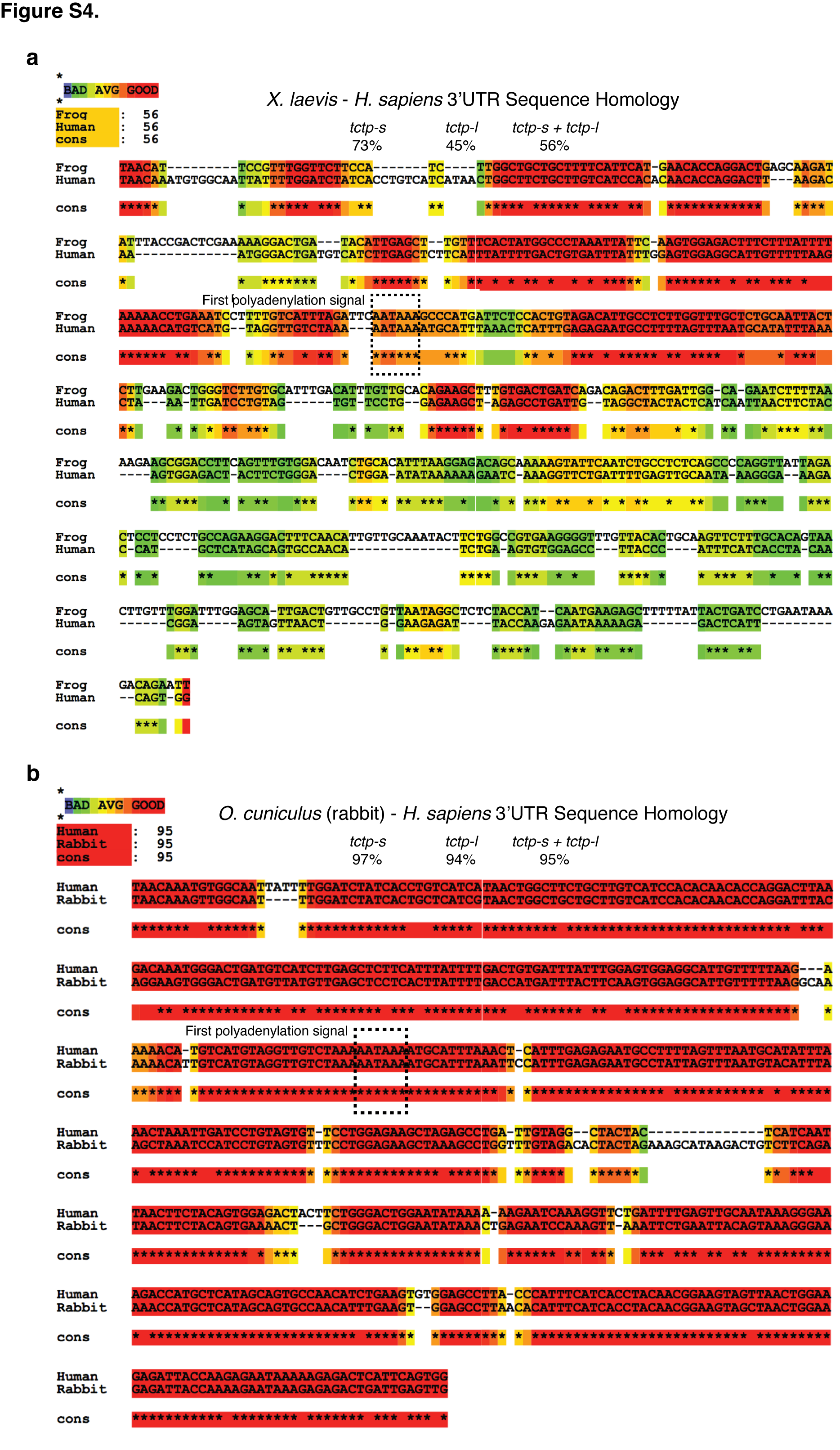

Supplement: FIGURE S4 — Poor sequence conservation between Xenopus laevis and Homo sapiens tctp-l. (A,B) Sequence alignment of Xenopus laevis, Oryctolagus cuniculus (rabbit), and Homo sapiens tctp 3′UTRs using T-Coffee. Both frog (73%) and rabbit (97%) show appreciable sequence homology relative to Homo sapiens upstream of the first polyadenylation signal. By contrast, the level of sequence conservation drops significantly in frog downstream of this motif (that is, in region corresponding to the unique stretch of the tctp-l 3′UTR), but remains nearly unchanged between rabbit and human. The boxed areas denote the location of the first polyadenylation signal (AATAAA). [file Image_4.TIF]

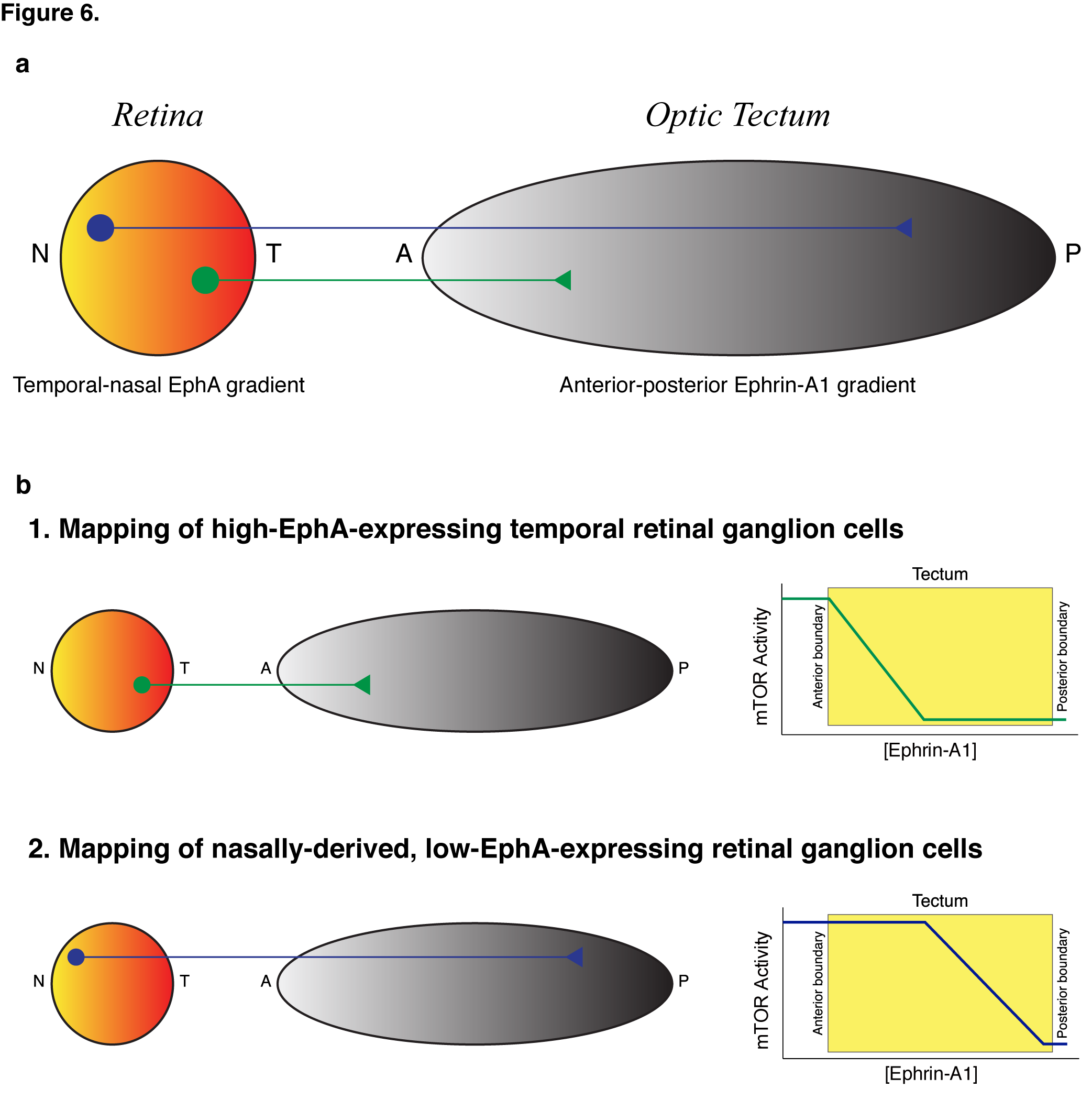

Supplement: FIGURE S5 — A model for how increasing Ephrin-A1 local concentrations can lead to a progressive inhibition of mTORC signaling across the naso-temporal axis during topographic map formation. (A) RGC axons originating from the temporal retina are high-EphA-expressing and project to the anterior-most, low-Ephrin-A1-expressing regions of the optic tectum. In turn, nasally-derived, low-EphA-expressing RGCs extend past the anterior optic tectum towards posterior (and high-Ephrin-A1-expressing) regions of the target field. This feature makes nasal processes unresponsive to low-Ephrin-A1-territories that temporal axons find restrictive, allowing nasal axons to project to the back of the optic tectum, where higher—and increasingly less permissive—Ephrin-A1 concentrations are found. It follows that low-EphA-expressing nasal RGC growth cones must have a higher threshold of response to Ephrin-A1, otherwise they would terminate precociously along the anterior-posterior axis. In other words, once inside the optic tectum, the relative progression of a RGC growth cone is dependent on the level of EphA receptor expressed on its surface, precisely matched to a repellent Ephrin-A1 counter-gradient—it is this molecular complement that defines the anterior-posterior coordinates of each discrete termination zone (McLaughlin and O’Leary, 2005). (B) Our findings suggest that a progressive inhibition of mTORC signaling promoted by increasing Ephrin-A1 local concentrations can potentially play a role in the fine modulation of this response, so that it occurs for each given growth cone only when its termination zone is reached and not before. TN, temporal-nasal axis; AP, anterior-posterior axis. [file Image_5.TIF]
